# Healthcare provider and medical cannabis patient communication regarding referral and medication substitution: the Canadian context

**DOI:** 10.1186/s42238-022-00141-0

**Published:** 2022-06-13

**Authors:** Alexis Holman, Daniel J. Kruger, Philippe Lucas, Kaye Ong, Rachel S. Bergmans, Kevin F. Boehnke

**Affiliations:** 1 Chronic Pain and Fatigue Research Center, Anesthesiology Department, University of Michigan, Ann Arbor, 24 Frank Lloyd Wright Drive, Ann Arbor, MI 48106 USA; 2 Population Studies Center, Institute for Social Research, University of Michigan, Ann Arbor, 426 Thompson St, Ann Arbor, MI 48106 USA; 3 Social Dimensions of Health, University of Victoria, 3800 Finnerty Rd, Victoria, BC V8P 5C2 Canada; 4 Tilray Canada Ltd., 1100 Maughan Rd, Nanaimo, BC V9X 1J2 Canada

**Keywords:** Medical cannabis, Medication substitution, Medical cannabis referral, Healthcare provider knowledge

## Abstract

**Background:**

Patients use medical cannabis for a wide array of illnesses and symptoms, and many substitute cannabis for pharmaceuticals. This substitution often occurs without physician oversight, raising patient safety concerns. We aimed to characterize substitution and doctor-patient communication patterns in Canada, where there is a mature market and national regulatory system for medical cannabis.

**Methods:**

We conducted an anonymous, cross-sectional online survey in May 2021 for seven days with adult Canadian federally-authorized medical cannabis patients (*N* = 2697) registered with two global cannabis companies to evaluate patient perceptions of Primary Care Provider (PCP) knowledge of medical cannabis and communication regarding medical cannabis with PCPs, including PCP authorization of licensure and substitution of cannabis for other medications.

**Results:**

Most participants (62.7%, *n* = 1390) obtained medical cannabis authorization from their PCP. Of those who spoke with their PCP about medical cannabis (82.2%, *n* = 2217), 38.6% (*n* = 857) reported that their PCP had “very good” or “excellent” knowledge of medical cannabis and, on average, were moderately confident in their PCP’s ability to integrate medical cannabis into treatment. Participants generally reported higher ratings for secondary care providers, with 82.8% (*n* = 808) of participants rating their secondary care provider’s knowledge about medical cannabis as “very good” or “excellent.” Overall, 47.1% (*n* = 1269) of participants reported substituting cannabis for pharmaceuticals or other substances (e.g., alcohol, tobacco/nicotine). Of these, 31.3% (*n* = 397) reported a delay in informing their PCP of up to 6 months or more, and 34.8% (*n* = 441) reported that their PCP was still not aware of their substitution. Older, female participants had higher odds of disclosing cannabis substitution to their PCPs.

**Conclusion:**

Most of the surveyed Canadian medical cannabis patients considered their PCPs knowledgeable about cannabis and were confident in their PCPs’ ability to integrate cannabis into treatment plans. However, many surveyed patients substituted cannabis for other medications without consulting their PCPs. These results suggest a lack of integration between mainstream healthcare and medical cannabis that may be improved through physician education and clinical experience.

**Supplementary Information:**

The online version contains supplementary material available at 10.1186/s42238-022-00141-0.

## Background

Canada legalized cannabis for medical use in 2001 and for adult recreational use in 2018 at the federal level (Understanding the new access to cannabis for medical purposes regulations 2016). Cannabis-based medicines have demonstrated effectiveness for treating a small number of conditions, including chronic pain, chemotherapy-induced nausea and vomiting, spasticity due to multiple sclerosis, and seizures in Dravet-Syndrome, Lennox-Gastaut Syndrome, and tuberous sclerosis complex (National Academies of Sciences E, and Medicine, et al. 2017; Devinsky et al. 2017, 2018; Thiele et al. 2021). In addition to these symptoms, observational studies show that patients use cannabis for a wider range of clinical needs (e.g., insomnia, anxiety, and depression) (Lucas et al. 2021). Patients also substitute cannabis for opioids, benzodiazepines, and antidepressants, as well as substances, such as alcohol and tobacco (Lucas et al. 2021; Lucas and Walsh 2017). Correspondingly, since the legalization of recreational cannabis in Canada, prescriptions for opioids, gabapentin, and pregabalin have decreased, especially among patients with public payer insurance plans (Dranitsaris et al. 2021).

The legalization of medical and recreational cannabis also correlates with its increased use for the management of various illnesses and symptoms. In a 2014 survey of 1000 Canadian patients diagnosed with a rheumatic condition, 4.3% reported ever using medical cannabis, and 2.8% reported current use (Ste-Marie et al. 2016). In 2018, after cannabis was legalized for non-medical adult use, 12.6% of respondents in a parallel survey of 1000 patients diagnosed with a rheumatic condition reported ever using medical cannabis, and 6.5% reported current use (Fitzcharles et al. 2020). However, most respondents obtained products from non-legal sources and did not know the THC content of the cannabis products they used (Fitzcharles et al. 2020), raising concerns about health effects due to potential contamination of unregulated cannabis products and the inability to provide repeatable therapeutic effects.

Pertinently, cannabis use and its substitution for medications and substances often occur without physician oversight (Fitzcharles et al. 2020). The majority (64%) of medical cannabis dispensary customers in Michigan surveyed in 2019–2020 used cannabis without the advice of their primary care provider (PCP), and 86% received medical cannabis authorization from a provider other than their PCP (Boehnke et al. 2021). Additionally, most participants lacked confidence in their PCP’s ability to guide their cannabis use and did not report their substitution of cannabis for other pharmaceuticals/substances to their PCPs (Boehnke et al. 2021). Taken together, these results suggest a lack of integration between mainstream healthcare and medical cannabis (Boehnke et al. 2021). PCPs are a vital front-line interface between patients and the healthcare system (Shi 2012); thus, improved understanding of patient-provider communication concerning medical cannabis use and substitution could inform strategies that maximize benefit and minimize harm.

In the present study, we investigated patient-provider communication concerning medical cannabis in the Canadian context, where a longstanding cannabis program is in place with a more mature market and national regulatory system. We surveyed Canadian medical cannabis patients about their perceptions of PCP medical cannabis knowledge and the nature of patient-provider communication regarding medical cannabis, including PCP authorization of licensure and substitution of cannabis for other medications. Given Canada’s long-standing medical cannabis program, we hypothesized that PCP authorization for medical cannabis licensure would be common. We also hypothesized that most patients would consider their PCPs knowledgeable about cannabis and disclose cannabis substitution to their PCPs. Lastly, we explored whether perceptions of PCP knowledge and competence regarding medical cannabis and disclosing substitution to one’s PCP varied by demographic factors (i.e., gender, race, age, educational level).

## Methods

### Setting and participants

Emails with an anonymous survey link were sent to 27,431 adult (aged ≥18 years) Canadian federally-authorized medical cannabis patients with physician recommendations for cannabis registered with Tilray and Aphria, two global cannabis companies, in May 2021. The survey link was active for seven days, and participants were incentivized to complete the survey with an opportunity to win one of three $1000 credits that they could use to purchase medical cannabis products from their Licensed Producer. Participants met the following inclusion criteria: completing electronic consent, capacity to consent for themselves, and fluency in English. Participants answered questions about demographics (gender, age, race/ethnicity, education), medical cannabis use and substitution of cannabis for other medications, and healthcare provider knowledge and ability to integrate medical cannabis into treatment. Overall, 2697 participants responded to the survey (1162 Tilray patients and 1535 Aphria patients), a 9.5% response rate. The survey went through ethics review and approval by Advarra, an independent Institutional Review Board service (ethics approval #Pro00050772).

### Measures

We used similar measures to those in the Michigan study and other medical cannabis studies that investigated substitution (Lucas and Walsh 2017; Boehnke et al. 2019a, 2021; Kruger et al. 2020; Kruger and Kruger 2019).

### Reasons for cannabis use and medical cannabis license authorization

Participants selected primary symptoms for which they used medical cannabis. They also indicated whether their PCP signed their medical cannabis license authorization. Participants answering “no” then selected whether they sought authorization from a secondary care provider. Those seeking authorization from secondary care providers indicated whether the secondary care provider worked in a clinic that specialized in medical cannabis, as well as whether the authorizing secondary care provider was involved in their care other than medical cannabis.

### Perceptions of healthcare providers regarding medical cannabis

Participants indicated whether they spoke with their PCPs about their medical cannabis use. Those who answered affirmatively rated their PCP’s knowledge about medical cannabis from *poor* to *excellent* and confidence in their PCP’s ability to integrate medical cannabis into treatment from *not at all confident* to *completely confident* on five-point Likert-type scales. Participants who sought authorization from secondary care providers rated these providers on the same measures. Responses were converted to continuous values (1–5) for statistical analyses.

### Substitution measures

We asked participants, “Did you find that medical cannabis helped you reduce or stop the use of…”, allowing them to choose from the list of medications from various drug classes based on previous research: i.e., alcohol, tobacco/nicotine, opioid prescription drugs, non-opioid prescription drugs, illicit substances (opioids, stimulants, psychedelics, etc.), other (Boehnke et al. 2021; Kruger and Kruger 2019). Those who disclosed substitution selected whether their PCP was aware of this substitution, with options for *no; yes, within a month; yes, within 6 months*; or *yes, but not for more than 6 months.*

### Statistical analyses

Descriptive analyses included frequencies, mean scores, standard deviations, and response ranges. Using independent samples t-tests, we compared various aspects of the physician-patient relationship, including (1) whether PCPs knew about drug substitution, (2) perceptions of PCP knowledge about medical cannabis by whether PCPs signed a medical cannabis recommendation, (3) confidence in PCP’s ability to integrate medical cannabis into their treatment by whether PCPs signed a medical cannabis recommendation, (4) whether participants sought a medical cannabis recommendation from a secondary care provider, (5) the secondary care provider’s knowledge about medical cannabis by whether they worked in a clinic that specialized in medical cannabis, and (6) and secondary care provider’s knowledge about medical cannabis by whether they were involved in health or medical care other than medical cannabis. We then compared perceptions of PCP and secondary care provider’s knowledge about medical cannabis using a within-sample t-test. Finally, we used binary logistic regression to investigate associations between demographic variables (age, gender, income, education) and any disclosure of substitution of cannabis for medications to a PCP. All analyses were conducted in SPSS (IBM, Armonk, NY).

## Results

### Sample characteristics

Our study sample (*N*=2697) was comprised of 50.1% (*n*=1,352) men and 49.1% (*n*=1,325) women with an average age of 54.3 years (Supplemental Table 1). Most (72.1%, *n*=1,945) had a technical degree or more education. Participants were primarily White (91.3%, *n*=2,463) and most used cannabis daily. The most common primary conditions for cannabis use were chronic pain (27.8%, *n*=750), arthritis (14.9%, *n*=402), anxiety (9.0%, *n*=242), insomnia (8.8%, *n*=238), other unlisted condition (7.7%, *n*=209), and fibromyalgia (6.4%, *n*=172), while the most common symptoms for cannabis use were chronic pain (66.6%, *n*=1796), anxiety (35.7%, *n*=964), insomnia/sleep disorder (34.6%, *n*=933), stress (24%, *n*=646), depression/low mood (22.3%, *n*=602), and headache/migraines (13.7%, *n*=369). Most participants (62.7%, *n*=1,390) obtained medical cannabis authorization from their PCP (Table 1). Of those who did not obtain authorization from their PCP, 74.7% (*n*=976) sought authorization from a secondary care provider, most of whom (88.6%, *n*=865) worked in a clinic that specialized in medical cannabis.

**Table 1:** Physician interactions with medical cannabis patients

	% Yes
Did your primary care provider sign your medical cannabis recommendation? (*n*= 2217)	62.7, *n* = 1390
Did you seek a medical cannabis recommendation from a secondary care provider? (*n*= 1307)	74.7, *n* = 976
Did your secondary care provider work in a clinic that specialized in medical cannabis? (*n*= 976)	88.6, *n* = 865

Data include patients who answered “no” to “Did your primary care provider sign your medical cannabis recommendation?”

### Perceptions of provider knowledge and confidence in ability to integrate medical cannabis into treatment

Participants who spoke with their PCP about medical cannabis (82.2%, *n*=2217) on average reported that their PCP had “good” knowledge of medical cannabis and were moderately confident in their PCP’s ability to integrate medical cannabis into treatment (Table 2). Overall, 38.6% (*n*=857) of participants rated their PCP’s knowledge about medical cannabis as “very good” or “excellent,” and 48.2% (*n*=1300) reported feeling “very confident” or “completely confident” in their PCP’s ability to integrate medical cannabis into treatment. Participants generally reported higher ratings for secondary care providers, with 82.8% (*n*=808) of participants rating their secondary care provider’s knowledge about medical cannabis as “very good” or “excellent.” Most secondary care providers (71.2%, *n*=695) were not involved in participants’ medical care other than authorizing medical cannabis.

**Table 2:** Perceptions of primary care provider (PCP) knowledge and confidence in ability to integrate medical cannabis into treatment

Questions asked	Percentage of respondents
**Proportion who spoke to PCP about medical cannabis use (*n*= 2697)**	82.2%, *n* = 2217
**How would you rate your primary care provider’s knowledge about medical cannabis? (*n*= 2217)**	
*Poor*	18.9%, *n* = 420
*Fair*	20.3%, *n* = 450
*Good*	22.1%, *n* = 490
*Very good*	21.7%, *n* = 482
*Excellent*	16.9%, *n* = 375
**Confidence in your primary care provider’s ability to integrate medical cannabis into treatment (*n*= 2697)**	
*Not at all confident*	18.1%, *n* = 488
*Somewhat confident*	15.4%, *n* = 414
*Moderately confident*	18.4%, *n* = 495
*Very confident*	28.4%, *n* = 765
*Completely confident*	19.8%, *n* = 535
**How would you rate your secondary care provider’s knowledge about medical cannabis? (*n*= 976)**	
*Poor*	1.2%, *n* = 12
*Fair*	3.3%, *n* = 32
*Good*	12.7%, *n* = 124
*Very good*	30.4%, *n* = 297
*Excellent*	52.4%, *n* = 511
**% Of participants whose secondary care providers were involved in healthcare other than medical cannabis (*n*= 976)**	28.8%, *n* = 281

Data include entire sample unless indicated

### Substitution for pharmaceuticals and other substances–with and without PCP knowledge or authorization

Overall, 47.1% (*n*=1269) of participants reported substituting cannabis for other pharmaceuticals or substances. The most common pharmaceutical substitutions were reported for opioid prescription drugs (18.0%, *n*=486) and non-opioid prescription drugs (17.5%, *n*=472) (Table 3). The most common substance substitutions were reported for alcohol (17.7%, *n*=478) and tobacco/nicotine (8.0%, *n* = 215). Of these, 34.8% (*n*=441) reported that their PCP was still not aware of their substitution, 34% (*n*=431) reported informing their PCP within 1 month, and 31.3% (*n*=397) reported delaying informing their PCP for up to 6 months or more (Table 3). Higher age and female gender predicted a greater likelihood of substitution disclosure to a PCP (see Table 4).

**Table 3:** Substitution of cannabis for other substances

Substances (***n*** = 2697)	%, ***n***
*Any*	47.1%, 1269
*Alcohol*	17.7%, 478
*Tobacco/Nicotine*	8.0%, 215
*Opioid prescription drugs*	18.0%, 486
*Non-opioid prescription drugs*	17.5%, 472
*Illicit substances (opioids, stimulants, psychedelics, etc.)*	1.9%, 52
*Other*	6.2%, 166
*None*	52.9%, 1428
Primary care provider knowledge of substitution (*n* = 1269)	
*No*	34.8%, 441
*Yes, within 1 month*	34.0%, 431
*Yes, within 6 months*	25.9%, 329
*Yes, but not for more than 6 months*	5.4%, 68

Data include the entire sample

**Table 4:** Demographic predictors of disclosure of substitution to primary care providers

	Unstandardized coefficients		Standardized coefficients	*t*	*P*
	*b*	SE	Beta		
(Constant)	0.458	0.055		8.34	<.001
Age in years	0.004	0.001	0.127	4.56	<.001
Male	− 0.071	0.027	− 0.074	− 2.65	.008
White	0.018			0.65	.514
Education level in years	− 0.021			− 0.88	.382
Annual household income in CAD	0.009			0.31	.759

Data include the entire sample. Values are results for logistic regression. The unstandardized coefficient represents the amount of change in the dependent variable due to a change of 1 unit of the independent variable. Standardized coefficients are normalized unit-less coefficients indicating effects strength. White race, education level, and income were not uniquely significant predictors of disclosure of substitution

### Associations between perceived PCP knowledge, confidence, and substitution disclosure

PCP knowledge about medical cannabis was rated higher if they signed a medical cannabis recommendation, *t*(2215) = 24.47, *p* < .001, *d* = 1.07. PCP knowledge about medical cannabis was rated lower if participants sought a medical cannabis recommendation from a secondary care provider, *t*(825) = 3.07, *p* < .001, *d* = 0.31. Confidence in PCP’s ability to integrate medical cannabis into treatment was rated higher if they signed a medical cannabis recommendation, *t*(2215) = 18.96, *p* < .001, *d* = 0.83. PCPs who knew about drug substitution were rated higher on knowledge about medical cannabis, *t*(2215) = 4.45, *p* < .001, *d* = 0.27. PCPs who knew about drug substitution were rated higher on confidence in ability to integrate medical cannabis into treatment, *t*(2695) = 10.47, *p* < .001, *d* = 0.55.

### Comparing PCPs and secondary care providers

Secondary care provider’s knowledge about medical cannabis was rated higher than PCP’s knowledge, *t*(710) = 45.75, *p* < .001, *d* = 1.72. Secondary care provider’s knowledge about medical cannabis was rated higher if they worked in a clinic that specialized in medical cannabis, *t*(710) = 12.39, *p* < .001, *d* = 1.25, but was not related to whether they were involved in health or medical care other than medical cannabis, *t*(974) = 1.39, *p* = .083, *d* = 0.10.

## Discussion

In this study, we examined the substitution of medical cannabis for pharmaceuticals, as well as doctor-patient communication patterns about substitution, among Canadian medical cannabis dispensary patients. As hypothesized, participants rated PCPs as being quite knowledgeable of cannabis (“good” on average), and the majority received PCP authorization for medical cannabis. However, our hypothesis about substitution disclosure was not confirmed, as many participants either did not communicate or delayed communicating for 6 months or more with their PCP about substituting cannabis for other medications or substances, such as opioids, alcohol, and tobacco (Boehnke et al. 2021). Overall, results suggest uneven integration between medical cannabis and mainstream healthcare, evidenced by limited doctor-patient communication concerning the substitution of cannabis for pharmaceuticals.

Given that Canada has a more mature regulatory framework than Michigan, it follows that comparatively more patients in Canada obtain licensure from their PCP (62.7% vs. 14%), and Canadian patients generally report that their PCP has better knowledge of medical cannabis (60.7% vs. 39% rated as “good” to “excellent”) and are more confident of their PCP’s ability to integrate cannabis into their treatment (48.2% vs. 18.9% rated as “very confident” or “completely confident”) (Boehnke et al. 2021). Indeed, in Canada, cannabis is legal medically and recreationally at a federal level versus in the USA, where cannabis is still classified as a Schedule I drug federally with varying laws governing its use in each state (Boehnke et al. 2019b).

These findings add to the medical cannabis and substitution literature (Boehnke et al. 2016, 2019a; Bradford and Bradford 2016, 2017; Bradford et al. 2018; Lucas et al. 2016, 2019; Reiman et al. 2017; Piper et al. 2017a; Corroon Jr. et al. 2017) and confirm Michigan results that substitution often occurs without physician oversight (Boehnke et al. 2021). Whereas substitution can have a beneficial impact, for instance by decreasing opioid use when cannabis is used as part of a harm reduction strategy (Lucas 2017), proceeding without physician involvement has associated risks. Indeed, adverse effects, such as illness recurrence, could result if patients substitute cannabis for disease-modifying drugs (Boehnke et al. 2021). Furthermore, open communication between patient and provider about medical cannabis could facilitate information sharing, as well as foster the therapeutic alliance and enable shared decision-making. Indeed, consistent with our study in Michigan (Boehnke et al. 2021), participants whose PCP authorized medical cannabis perceived their PCP to have better medical cannabis knowledge and greater confidence in their ability to integrate cannabis into treatment. In addition, a recent survey reported that 24% of participants considered health professionals to be their most influential information sources about cannabis, and these participants were less likely to believe false claims about cannabis (Ishida et al. 2020). Lastly, a qualitative study of older adults showed that most participants wanted to discuss cannabis with their physicians despite expressing concerns about provider openness and knowledge about medical cannabis (Bobitt et al. 2019). These studies highlight the importance of the doctor-patient relationship in maximizing benefits and minimizing risks associated with medical cannabis use.

Our results showed that older and female participants were more likely to disclose cannabis substitution. Polypharmacy is prevalent among older individuals (Dagli and Sharma 2014); thus, concerns surrounding drug-drug interactions may prompt older individuals to discuss medication use with their physicians. Furthermore, older individuals have more health issues and visit the doctor more frequently (Institute of Medicine 2008), providing greater opportunity for conversation. Additionally, the elderly may be more susceptible to adverse effects from THC (Kaufmann et al. 2020) and may be more cautious in guiding their own use. Women have overall higher healthcare utilization than men (Bertakis et al. 2000; Ladwig et al. 2000) and are more likely to use complementary and alternative medicine (Zhang et al. 2015) both of which may increase the likelihood of speaking with providers about medical cannabis use. Furthermore, men generally have a higher prevalence of cannabis use (Wall et al. 2019; Compton et al. 2016) and may feel less need to disclose due to their knowledge gleaned from historical use. However, this reporting difference based on gender is intriguing in light of a recent survey study showing that women in Illinois felt less supported by specialist providers in their use of medical cannabis compared with male respondents (Bruce et al. 2021). Additionally, stigma regarding cannabis use may be exacerbated for females with children (Dahl and Sandberg 2015), with some women expressing difficulty reconciling internal and external expectations of motherhood with cannabis use (Dahl and Sandberg 2015; Hathaway et al. 2011). More research on the interface between gender and substitution is needed to better contextualize these findings.

The reason for the communication gap about medical cannabis in healthcare is likely multifaceted, creating barriers for both patients and physicians to open lines of communication. One explanation for the delay in communication about medical cannabis between patient and provider could be due to a lack of regular PCP visits. While this may be a contributing factor, there are other important considerations. For instance, in addition to patient concerns about PCP knowledge and ability to integrate medical cannabis into treatment, cannabis’s historical criminalization has caused patients to fear stigma, legal, or professional ramifications—all of which may discourage open communication about medical cannabis (Boehnke et al. 2021; Piper et al. 2017b; Bottorff et al. 2013; Lau et al. 2015a, b). A recent survey study of cancer patients in Italy showed that although 34% of respondents had considered using medical cannabis, only 11% considered contacting their physicians to obtain more information (Cortellini et al. 2019). While 81% of respondents had heard about medical cannabis, only 2% reported hearing about it from their physician (Cortellini et al. 2019). Likewise, many physicians remain reluctant to use medical cannabis in clinical practice. A recent meta-analysis including data from five countries with different medical cannabis regulatory systems showed that although physicians experience a substantial rate of patients inquiring about medical cannabis, there is a wide variability in their openness to prescribing, authorizing, and/or providing it (Ronne et al. 2021). Beliefs about medical cannabis appear to depend in part on experience, with providers who have more experience authorizing medical cannabis believing more in its beneficial effects and feeling less concerned about its adverse effects than those with no experience (Ronne et al. 2021). In addition, provider knowledge about medical cannabis also appears to affect provider attitudes. A recent survey study of Italian medical oncologists and palliative care physicians showed that while only a minority (14%) of respondents could name the law governing medical cannabis use, this knowledge correlated with a greater probability of prescription (Filetti et al. 2021). Furthermore, there was a gap in respondent knowledge, with a significant minority (31%) of respondents reporting not having enough information to distinguish the side effect profiles of medical cannabis and opioids and only 9 of 140 respondents reporting medical cannabis side effects in their patients (Filetti et al. 2021).

Notably, this study extends beyond prior work to consider the role of secondary care providers. Over one-third of current participants approached secondary care providers working in specialized medical cannabis clinics, whom they rated higher on measures of knowledge and ability to integrate cannabis into treatment. Although this greater perceived knowledge is unsurprising given the specialization, this highlights an area where continued improvement is needed, especially as providers in medical cannabis clinics may potentially have a higher degree of conflicts of interest. Ideally, even if patients sought advice on cannabis use from a secondary provider, patients would obtain a recommendation from their PCPs, who are presumably more familiar with patient medical histories, have established relationships, and have continued involvement in their healthcare (Bobitt et al. 2019). In addition, without continuous visibility on important treatment changes undertaken by their patients, including changes in prescription drug use, providing appropriate care becomes more challenging. Further understanding of the secondary provider relationship is also needed, as 71.2% (*n* = 695) of participants reported no continued involvement of their secondary provider in their medical care, suggesting that these participants still may need ongoing support in optimizing cannabis use.

While Canada has a longstanding medical cannabis program, our results indicate that gaps persist regarding its integration into mainstream healthcare systems. Increasing provider education concerning medical cannabis could help address this gap. Indeed, a small minority of medical schools (9%) include medical cannabis curriculum content (Evanoff et al. 2017). This lack of formal education combined with the lack of robust cannabis clinical trials literature to guide physician recommendations creates a situation in which patients are largely on their own to make challenging decisions. However, by using harm-reduction and benefit-maximization strategies, physicians can help guide safe patient use of medical cannabis. Using a combination of administration routes, as well as a “low and slow” dosing technique, for instance, can help physicians tailor their treatment plans to patient needs (Boehnke and Clauw 2019; MacCallum and Russo 2018). Education about these principles, as well as clinical experience applying them to clinical care, would improve physicians’ abilities to engage in shared decision making with their patients about medical cannabis (Boehnke et al. 2021; Ronne et al. 2021).

## Limitations

Our study has various limitations. The most significant limitation is the low response rate (9.5%) and predominantly White, educated sample. This may reflect selection bias, constraining the generalizability of our results. Second, our results may be affected by recall bias since we did not include any objective measures on substitution, and questions about PCP/secondary care provider attitudes were not asked during any visits. Third, the perceptions of PCP/secondary care provider knowledge and ability to integrate medical cannabis into treatment may not be reflective of the true abilities of those providers. For instance, a secondary care provider may appear more confident if they are marketing a product, potentially enhancing perceptions of their expertise.

## Conclusions

This study examined patient-provider communication patterns concerning cannabis use and substitution in Canada. Results suggest that patients often substitute cannabis for other medications without PCP guidance. The lack of integration between mainstream healthcare and medical cannabis could likely be improved through increased physician education and clinical experience. Although opportunities for improvement remain, our results also demonstrate how Canada’s stable federal policy around medical cannabis over the past 20 years has shifted clinical decision making towards a more accepting policy around cannabis, which could inform research in regions with more stringent cannabis policies. Future studies should investigate strategies for effectively involving PCPs in patient care around medical cannabis with specific focus on substitution and harm reduction practices.

## Supplementary Information

**Additional file 1: Table S1.** Demographics of survey respondents (*N* = 2697).

## Data Availability

Data and survey available upon reasonable request.
