# Supplementary material for: Healthcare provider and medical cannabis patient communication regarding referral and medication substitution: the Canadian context
Source: J Cannabis Res. 2022 Jun 13;4:32. doi: 10.1186/s42238-022-00141-0 (PMC9195481; doi:10.1186/s42238-022-00141-0)
Supplement: Supplementary file 1 — Additional file 1: Table S1. Demographics of survey respondents (N = 2697). [file 42238_2022_141_MOESM1_ESM.docx]

**Table S1. Demographics of survey respondents (*N* = 2697)**

| **Descriptive** | **Value** |
| --- | --- |
| **Gender** |  |
| *Men* | 50.1%, *n* = 1352 |
| *Women* | 49.1%, *n* = 1325 |
| *Other* | 0.4%, *n* = 10 |
| *Unknown* | 0.4%, *n* = 10 |
| **Age in years (*M, SD*, range)** | 54.3, 14, 20-90 |
| **Education** |  |
| *Less than high school* | 4.2%, *n* = 114 |
| *High school or equivalent* | 23.7%, *n* = 638 |
| *Technical and non-university degree* | 41.5%, *n* = 1118 |
| *University degree* | 22%, *n* = 593 |
| *Graduate degree* | 6.7%, *n* = 182 |
| *Doctorate or professional degree* | 1.9%, *n* = 52 |
| Races/ethnicities (inclusive) |  |
| *White* | 91.3%, *n* = 2463 |
| *Hispanic* | 1.0%, *n* = 27 |
| *Asian* | 1.1%, *n* = 29 |
| *South Asian* | 1.4%, *n* = 37 |
| *Black* | 1.3%, *n* = 36 |
| *Aboriginal/First Nation* | 2.5%, *n* = 67 |
| *Metis* | 2.4%, *n* = 64 |
| *Other* | 2.4%, *n* = 70 |
| **Current relationship status** |  |
| *Married* | 57.1%, *n* = 1541 |
| *Widowed* | 4.0%, *n* = 109 |
| *Divorced* | 9.7%, *n* = 262 |
| *Separated* | 3.2%, *n* = 86 |
| *In a domestic partnership or civil union* | 9.7%, *n* = 262 |
| *Single, but cohabiting with a significant other* | 3.9%, *n* = 106 |
| *Single, never married* | 12.3%, *n* = 331 |
| **Frequency of cannabis use** |  |
| *Days per week (M, SD, range)* | 6.5, 1.3, 1-7 |
| *Times per day (M, SD, range)* | 2.9, 2.1, 1-10 |
